# Exploring the Perceptions of the Ageing Experience in Singaporean Older Adults: a Qualitative Study

**DOI:** 10.1007/s10823-020-09414-8

**Published:** 2020-10-09

**Authors:** F. Shiraz, Z. L. J. Hildon, H. J. M. Vrijhoef

**Affiliations:** 1 Saw Swee Hock School of Public Health, National University of Singapore, Tahir Foundation Building, Singapore, 117549 Singapore; 2 National University Health System, Singapore, Singapore; 3 Johns. Hopkins University, Center for Communication Programs, Baltimore, MD USA; 4 Department of Patient & Care, Maastricht University Medical Center, Maastricht, the Netherlands; 5 Department of General Practice and Chronic Care, Vrije Universiteit Brussels, Ixelles, Belgium; 6 Panaxea b.v, Amsterdam, the Netherlands

**Keywords:** Asian older adults, Ageing, Ageing experiences, Biopsychosocial health, Health perceptions

## Abstract

Understanding older adults perceptions of health and adaptation processes to ageing can allow for more culturally aligned services and better targeted care. The aim of this exploratory qualitative study was to examine older adults perceptions of physical, psychological and social health and further understand the processes of adaptation and self-management of these health perceptions. Semi-structured in depth interviews (IDI) were conducted with ethnically diverse older adults in Singapore, aged 60 and above. Participants were asked open ended questions about their physical health, psychological health and their current social health and relationships. They were also asked methods of adaptation to these age related changes. In total, forty participants were interviewed. A thematic analysis identified five main themes when exploring perceptions of physical, psychological and social health. These included; 1) Slowing down 2) Relationship harmony 3) Financial harmony 4) Social connectedness and 5) Eating together. Adaptation and self-management of these health perceptions included six additional themes; 1) Keep moving 2) Keep learning; where continued self-determination and resilience was a key method in adapting to negative thoughts about declining physical health 3) Adopting avoidant coping behaviours 4) ‘It feels good to do good’; where finding meaning in life was to help others 5) ‘Power of Prayer’; which highlighted how older adults relegated responsibilities to a higher spiritual power 6) Social participation; which included engaging in community and religious social activities that all contributed to self-management of older adults psychological health and social health. In conclusion, our study highlighted specific cultural nuances in older adults perceptions of health, particularly psychological and social health. These findings can help develop more targeted intervention programmes and better methods of measuring older adults health, which can assist with the global ageing phenomena.

## Introduction

The worldwide shift in the epidemiology of ageing has created global phenomena where we will soon have a higher prevalence of older people than children and more people at extreme old age than ever before. A report by the United Nations (2019) highlighted that globally there were 703 million older persons aged 65 or over in 2019 with Eastern and South-East Asia being home to the largest number of the world’s older population (260 million), followed by Europe and Northern America (United Nations 2019). In addition, between 2019 and 2050 it has been reported that nine out of the ten countries with the largest increase in the share of older persons in the world will be in Eastern and South-East Asia (United Nations 2019). The UN report highlighted that in Eastern and South East Asia the ageing population will grow from 261 million in 2019 and to 573 million persons aged 65 years or over by 2050, with the largest increase foreseen in the Republic of Korea followed by Singapore (United Nations 2019: 7). This means Eastern and South-Eastern Asia will be home to the largest share (37%) of the world’s older population in 2019 and this is expected to remain so in 2050. With this increasing prevalence, optimising older adults health in Asia is at the core of striving towards improving the quality of life of this older population.

Singapore is recognising this change within its demographic and in 2015, the Ministry of Health in Singapore unveiled a $3 billion national plan to help Singaporeans age confidently and lead active lives (Ministry of Health Singapore 2016). This national initiative for successful ageing set aside a significant part of its budget for a National Innovation Challenge on Active and Confident Ageing to catalyse research related to ageing (Ministry of Health Singapore 2016). Corresponding with this initiative the Community for Successful Ageing (ComSA) programme of research was set up by the Tsao Foundation who aimed to provide a holistic biopsychosocial (BPS) community-wide, ground up approach to deliver an integrated system of comprehensive programmes for older people (Tsao Foundation 2015). As part of this programmatic approach, a key initiative was to identify ‘vulnerable’ older people who suffer from increased biopsychosocial health risk and to try and better engage them in community based services. Thus it was important to begin to understand the needs and health perceptions of older adults to allow for programs to be tailored to help to stave off vulnerability, improve quality of life (QoL) and keep people ageing in place for longer.

### Perception of Health in Older Adults

The World Health Organisation’s (WHO) early definition of health encompassed health as a state of *‘*complete physical, mental and social well-being and not merely the absence of disease or infirmity’ (WHO World Health Organisation 2006). This definition was a positive shift towards seeing one’s health as a holistic entity, incorporating physical, mental as well as social aspects of health. However, in 2011 Huber and colleagues challenged this definition highlighting the need for more emphasis on social and personal resources of an individual as well as physical capacity (Huber et al. 2011). They rallied for redefining health with the aim for moving from the WHO’s static formulation towards a more dynamic one based on ‘the resilience or capacity to cope and maintain and restore one’s integrity, equilibrium, and sense of wellbeing in terms of the ability to adapt and to self-manage’ (Huber et al. 2011). This was further conceptualised as ‘positive health’ arguing that there is the need for a more dynamic description of health that highlights the human capacity for resilience and for coping with new situations (Huber et al. 2016: 10).

Huber et al. (2011, 2016) re-conceptualisation of health has implications within the ageing literature. As we know, older people themselves have demonstrated the phenomenon dubbed the ‘paradox’ of ageing, drawing consternation at their unexpectedly consistent and relatively high levels of well-being and satisfaction at a population level (Gana et al. 2013; Staudinger 1999) despite having functional and/or physical health limitations. Studies have shown that older adults will perceive themselves as having aged successfully regardless of their clinical health status (Cho et al. 2015; Pruchno et al. 2010; Jeste et al. 2013). This phenomenon is often explained by the selective optimization and compensation (SOC) model, (Baltes and Baltes 1990; Freund and Baltes 1998) which describes how older adults capitalise on their internal and external resources and coping mechanisms, integrating both psychological and social resources to help buffer potentially harmful stressors that can arise from disease and other age related factors (Freund and Baltes 1998, 2002; Baltes and Lang 1997; Baltes and Baltes 1990). The SOC theory of ageing and Huber’s reconceptualization of health show positive shifts away from viewing health as simply an ‘absence of disease’. Both theories help to evolve the concept of what is means to age successfully. However, both stances are underpinned in evidence that has been conducted within the US and Europe (Baltes and Baltes 1990; Freund and Baltes 1998; Huber et al. 2011, 2016) thus to advance on the issues of global ageing, further work is required to understand how older adults around the world perceive their health and ageing. The potential of cultural nuances in perceptions of the ageing process can differ significantly across different countries (Fung 2013). This may be due to a number of factors including differences in cultural beliefs, social norms, and internalized cultural values which may play a role in the cultural differences in perceptions of ageing (Fung 2013).

### Cross Cultural Ageing

Triandis (1989) proposed that there are two distinct cultures across the world- individualist and collectivist cultures*.* Triandis (1989) highlighted that individuals from individualist cultures (sometimes referred to as independent cultures) will have a unique set of internal traits, values, and emotions contributing towards autonomy and differentiation from others, where the self is neither overly connected to nor influenced by others (Triandis 1989; Markus and Kitayama 1994, 1998). It is theorised that individualist cultural views are more dominant within ‘West’ (North America and Europe). In contrast, in collectivist cultures individuals’ social behaviour and internalised values will be largely determined by goals, attitudes, and values that are shared with some collectivity, i.e. group of individuals (Triandis 1989; Markus and Kitayama 1994, 1998). These differences may come to impact older adults perspectives on ageing and their adaptation strategies.

One explanation of the differences between individualist and collectivist cultures has been due to the religious heritage for many South East Asian countries i.e. Hinduism, Buddhism, Shintoism, and Confucianism (Triandis 1989; Markus and Kitayama 1994, 1998). These religions do not regard the self as being distinct from others instead the focus is on the self in relation to and interdependent with other people (Ho 1998; Markus and Kitayama 1994). Thus, in contrast to the ‘western’ orientation of autonomy and differentiation, the collectivist (often of Asian descent) orientation focuses on connectedness to and harmony with others (Ingersoll-Dayton et al. 2001). For example, in many Chinese societies the family has been central to the social organization for thousands of years (Cheng and Chan 2006) and there is a long history in which the Chinese family functions as a close-knit social unit from which its members draw on each other’s resources for meeting psychological, social, and physical needs. This is significant when exploring the perceptions of health and ageing within South East Asian countries such as Singapore, where confucian teachings may still dominate particularly amongst the Singaporean Chinese population.

Confucian teachings include the concepts of filial piety, or Xiao, which regulates the expectation of relationship between children and their parents (Chow 2001; Cheng and Chan 2006). Filial piety includes showing respect, being obedient, honoring or promoting the public prestige of the parent and the ancestors, living with the parent (or staying close if co-residence is not possible), taking care of the parent whether healthy or sick, and avoiding injury to self because the body belongs to the parent, among others (Chow 2001). These factors may play a key influence in how older adults within Singapore perceive ageing, particularly regarding their psychological and social health.

### Purpose of the Study

This exploratory qualitative study aimed to: 1) examine older adults perceptions of their physical, psychological and social health; and 2) understand how older adults choose to adapt and to self-manage, in the face of these social, physical and emotional challenges.

## Methods

This study was an exploratory qualitative research design, grounded within interpretative approaches using in-depth interviews to provide access to accounts of how respondents perceive, understand and talk about the ageing process. The present study was the second phase of a larger multi-phase mixed methods study (The Community for Successful Ageing in Singapore) which aimed to better understand the health-related issues of older adults in Singapore and implement and develop appropriate holistic measurement tools, to identify hard to reach and vulnerable older adults in the community (Hildon et al. 2018). The Community for Successful Ageing (ComSA) programme was part a national initiative and is a community-wide, grounds up approach in the Whampoa neighbourhood of Singapore (approx. 33,000 residents of whom a third is aged 50 and above and 3600 crossed the age of 65) to co-build an integrated system of comprehensive programmes, services and enabling environments for health, wellbeing, personal growth and participation over the life course**.** The ComSA programme has been coordinated by the Tsao Foundation since 2014 (Tsao Foundation 2014).

This study presents the findings from the qualitative phase of the ComSA research study.

### Subjects

The study population comprised of Singapore residents (Singapore citizens and permanent residents) aged 65 and above who were living in Singapore. We used purposive maximum variation sampling strategy to allow for ethnic diverse participants and to capture diversity across age groups.

All participants approached had participated in a phase one of this study (Hildon et al. 2018). Participants were contacted by mail with the option to opt out by returning a self-addressed envelope or by door-to-door knocking. Following informed consent, semi-structured interviews were conducted.

Interviews were held between October 2015 and December 2015 and were conducted in the homes of participants in language or dialect of participant’s choice. Our bilingual research team were able to conduct interviews in, English, Malay Mandarin, and Cantonese. In addition, if requested, interviews were also conducted in local Chinese dialects which included Hokkien and Teochew.

The research team consisted of one gerontology trained research assistant, two senior postgraduate research fellows and one senior assistant professor.

### Data Collection

Participants were asked open ended questions about their physical health (e.g. Can you tell me how you feel you may have slowing down in last 12 months?), psychological health where older adults were specifically asked questions about their emotional wellbeing which included questions related to mood, worries and anxieties (e.g. Can you tell me about any current roubles, stress or worry you are experiencing?) and their current social relationships (e.g. Can you tell me about your relationship with your partner and family members?). Additional prompts and probing questions were used to gain a deeper understanding into these three specified areas.

The interviews lasted approximately 1 to 1 ½ hours and were digitally recorded to facilitate later analysis. Field notes and reflexive journaling was undertaken in English immediately after each interview. Audio-recordings were reviewed alongside reflexive notes to aid transcription using an expanded notes approach as proposed by Halcomb and Davidson (2006). This approach uses the following six steps to assist with qualitative data management.

Step 1: Audio taping of interview and concurrent note taking. Step 2: Reflective journaling immediately post interview. Step 3: Listening to the audio and revising field note observations. Step 4: Preliminary content analysis, where once researchers were confident that their field notes accurately represented the interactions that occurred in each interview, the process of content analysis was used to elicit common themes between interactions. Step 5: The preliminary content analysis was reviewed by a second research team member who had not previously been involved in the data collection thorough review of field notes. This task facilitated testing of the audit trail and validation of the development of themes from the data. Step 6: Thematic review: This final stage involved reviewing the secondary content analysis, making any necessary change to established themes and re-listening to the audio recordings to identify illustrative examples with which to demonstrate the meaning of the themes from the participants’ perspectives (Halcomb and Davidson 2006: 41).

All reflective notes, field note observations and illustrative examples of verbatim text which demonstrated the meaning of the themes, were recorded using Microsoft Word. This process was completed by the research team member who had conducted the interview, with all data translated into English. No repeat interviews were carried out and transcripts were not returned to participants for comments or corrections. Data saturation was discussed within the team and there was consensus that data saturation for the purpose of research objective had been achieved.

The data collection team consisting of three senior qualitative researchers (ZH, FS ZL) with PhD; one Masters in Gerontology (CL) researcher and one PhD student (SA). ZL, CL and SA were fluent in Mandarin and various Mandarin dialects. ZL and CT also provided Malay interpretation. The IDI’s were conducted with participants who had no prior relationship with the researchers. The research team was female.

### Data Analysis

All interview audio recordings were manually transcribed into Microsoft Word document form by the research team member who had conducted the interview. Each research team member deductively analysed their transcripts utilising the following pre-defined framework: Biological/physical Health (B). Emotional /psychological health (P). Social Health i.e. current social relationships (S). Adaptation and coping mechanisms with age-related adversities. New generated themes and sub-themes were also identified (inductive analysis) using an iterative process. Patterns, associations, concepts and explanations in the coded data were allocated quotes and or extracts to support each final theme. The leading author was involved in reviewing all the deductively analysed transcripts and inductively used thematic analysis to elicit additional themes or unexpected findings through further coding and categorisation of data.

The thematic analysis involved the following four components: 1) Familiarizing of data. 2) Identifying codes and themes. 3) Coding the data. 4). Organizing the codes and themes (Braun and Clarke 2006). To facilitate analysis, generated themes were consistently discussed with all research team members (FS, ZH, CT, SA, ZL BV) to reach consensus on the interpretation of the data. All relevant data was mapped and reported accordingly. Nvivo 11.0 software assisted with the organisation of the data.

### Ethical Consent

This study was registered and approved (B15–105) by the National University of Singapore institution review board in 2015. Informed consent procedures were implemented in this study where all participants provided written consent or thumbprint if unable to provide a signature. Participants who had shown to have poor cognitive functioning were excluded from this study. A pseudonym was provided for all IDI and extracts.

## Results

The results from this study highlighted the perceptions and adaptations of physical, psychological and social health of older adults within an Asian context (Singapore). We purposively sampled 200 older adults from an elders community survey conducted in Singapore between August to October 2014 (Hildon et al. 2018). Thirty-one opted out via post. The remaining 169 participants were approached via door-to-door knocking. Sixty-four did not respond and sixty-five had declined. Non-responders declined to give reasons for not participating. In total forty (13 male, 27 female) agreed to take part in in-depth interviews (IDI).

One IDI participant declined to be audio-recorded so in-depth field notes were collected.

The age of participants ranged between 60 and 87. Sixty-five per cent identified their.

ethnicity as Chinese, 17.5% Malay, 12.5% Indian and 5% as other (Table 1). Place of birth for participants was not recorded, however all participants identified themselves as a Singapore Resident (Singapore Citizens and Permanent Residents of Singapore).

**Table 1:** Age, Gender and Ethnicity of in-depth interview participants, (n = 40)

Participant Characteristics			
Characteristic	Female	Male	Total
Gender	27	13	40
Age Range (years)			
60–74	22	8	30
75–84	5	2	7
85+	1	1	2
Ethnicity			
Chinese	19	7	26
Malay	4	3	7
Indian	3	2	5
Other	2	0	2

### Thematic Analysis

**Fig. 1:**
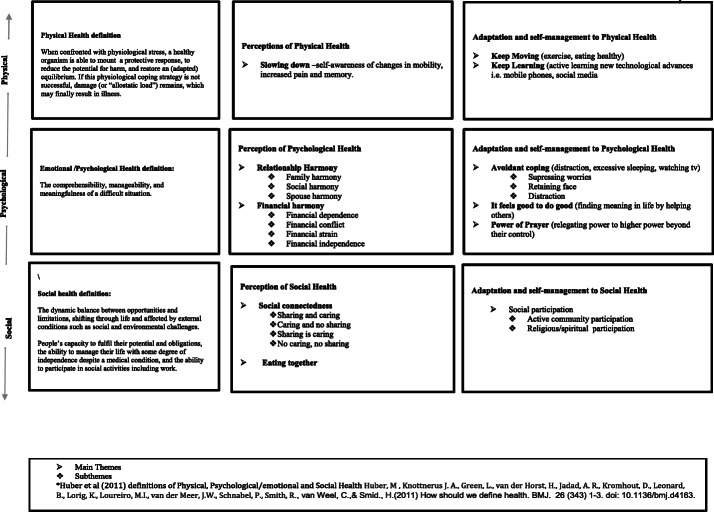
i) Huber’s definitions of Health Domains (Physical, psychological and social Health), ii) Qualitative themes of older adults perceptions of physical, psychological and Social Health iii) Qualitative themes of Adaptation and self-management strategies to physical, psychological and Social Health (*n* = 40)

Figure 1 illustrates a) The health framework used to classify older adults perceptions of physical, psychological and social health. b) Themes and subthemes of older adults perceptions of physical, psychological and social health and c) Themes and Subthemes demonstrating adaptation and self-management strategies. Herein we describe each main theme and subtheme.

### Perceptions of Physical Health

#### Slowing Down

Many older adults expressed functional declines which included; changes in mobility, increased pain and changes in short-term memory. However, they perceived their physical health changes as part of the ageing process. This was particularly apparent in participants aged 70 and above who shared a sense of acceptance of this ‘slowing down’ process.

“This is the facts, when you are getting old you don’t have the energy, and you are slowing down, so you cannot work too hard. You are not like a tiger, not so strong, no such thing, so you must take care of yourself. When you are getting old there is a lot of sickness coming, because like a car you get rusty. Of course, I worry about my health, because one day I may fall that is very serious, so I try and be very careful”. Female, 70, Chinese.

The extract highlighted an insightful view into this participant’s perception of ageing. She used several metaphors describing herself as a rusting car and not being as strong as a tiger which allowed her to normalise the ageing process. We saw similar forms of acceptance from other participants where one participant stated “Old already is like that” Female, 73. Chinese. Thus there was an overall acceptance that slowing down was just a part of the ageing process.

#### Adaptation and Self-Management of Physical Health

Two key themes were identified as the main adaptation and self-management methods when older adults were asked questions related to their adaptation and self -management strategies towards physical health. These themes were to; Keep Moving and Keep Learning.

### Keep Moving

Adapting to these physical health changes included adopting a resilient attitude but also engaging in behaviours that were believed to assist with the management of their physical health. This included the concept of ‘Keep Moving’ where older adults reported engaging in physical activities, (tai chi, walking, jogging) where they believed engaging in physical activity would keep them healthy and stave away diseases related to ageing. For older adults physical activity was seen as part of a healthy lifestyle that they linked to the prevention of disease and something that sustained independence.

“You stay healthy when you sweat and do more work [referring to exercise], so you won’t have these sort of diseases” Female, 70, Chinese.

### Keep Learning

Similarly, the theme of Keep Learning highlighted how learning new skills helped older adults maintain a ‘sharp’ and ‘active’ mind particularly when keeping up with technological advances such as social media applications (i.e. Facebook, Facetime). Using such social media applications gave many of the older adults the confidence to learn new things as they felt they were still connected with modern society. For some older adults, learning new technologies allowed them to stay engaged with their children and grandchildren, thus motivating their desires to learn about social media technology. For example; Mdm K was a 61, Chinese female who believed her memory was “still sharp as ever” due to her constant communication with her two young grandchildren. She guided them in their school work and tutored them in their Chinese language. Mdm K used a smartphone and talked of engaging in social media applications to help stay in touch with her children and to stay updated on current news and social affairs. This helped with her feeling connected with society and to ‘stay young’.

#### Perceptions of Psychological Health

When older adults were asked about their psychological health they were asked several questions about everyday ‘troubles’ which in Singapore refers to anxieties, worries and factors that contributes to low mood. Older adults reported two key themes: Relationship Harmony and having Financial Harmony.

### Relationship Harmony

The importance of relationship harmony dominated older adults narratives. Older adults reported experiencing more positive emotions when interacting with their social partners which included; their family, spouse and friends. Moreover, perceptions of positive relationship harmony allowed older adults to experience greater life satisfaction and happiness. This ‘harmony’ consisted within the following three typologies: family harmony, social harmony and spouse harmony.

#### Family Harmony

For many older adults their family members were especially important to achieve positive psychological health. They described how having harmony within their families allowed them to not only have good emotional support but they shared the belief that they had achieved a life goal by raising harmony within the family.

“I always have that support, I do not mean financial support but I have close siblings, a good family, grandchildren, I feel blessed. I am not financially blessed but mentally and physically”. Female, 62. Chinese.

#### Social Harmony

Older adults expressed the desire to achieve relationship harmony not only in their relationships but also amongst individuals in their social network. The negative emotional impact of family disputes and/or a family illness was consistently reported.

“Every home has their problems, for example my sister has problems with her daughter, and her son. My younger sister also has family problems. I cry (for them), after crying I will feel better”. Female, 60. Malay.

What this quote highlighted was a form of interdependency in emotions where the emotional distress experienced by family members was simultaneously experienced by the older adult. This demonstrated an example of an interdependent family, where an allegiance to one another was part of the core values of the family.

#### Spouse Harmony

This subtheme of spouse harmony was particularly dominant amongst the female older adults and was not a singular case. In our study, some female participants reported the frequent verbal abuse received from their partner over the years, however due to cultural or religious affiliations they chose to remain “silent” in order to prevent social embarrassment. There was a consistent notion that such stresses or ‘problems’ were personal and best with dealt alone. Many felt they were unable to trust or disclose this information to family members. Other participants identified similar circumstances where they had a desire to leave their relationship but due to family and social expectations continued to stay married, which had significant impact on their emotional health. The below extract highlighted the social and cultural expectations and difficulties some older adults found in leaving their marriage.

“I feel it is a big sin (referring to leaving husband). I cannot say (how she really feels) you see how sad I am. Thirty years I have looked after (my) children. People won’t believe what I tell them because my husband seems so nice. People won’t believe me, but what can I do? I’m old already, handicapped already, so I have to keep quiet and shut my mouth.” Female, 66. Malay.

### Financial Harmony

The second main theme identified by older adults as having both positive and negative impact on their psychological health was about having financial harmony. To have financial harmony was recognizing the lack of concern about financial restrictions in their later lives. For many older adults financial worries were a key life stressor, which had a significant impact on their psychological health. This theme consisted of four distinct subthemes; financial dependence, financial conflict, financial strain and financial independence. Financial dependency, conflict, and strain in particular were identified as having a negative impact on older adults’ psychological health.

#### Financial Dependence

The older adults highlighted how their children were often the main source of financial support. They reported feeling both content and conflicted with this source of support. On the one hand they had a desire for financial independence but in some cases often having no choice but to be dependent on family to provide financially for them.

“I am not rich but I am well provided. I think among the family you should always take care of each other first. If they can’t help you, only then should you go and seek help”. Female. 62. Chinese

#### Financial Conflict

This subtheme represented the conflict between expectations from the older adults vs expectation from their financial providers who were more than often their children. These sources of financial conflict were reported to have significant impact on older adults psychological wellbeing, often resulting in withdrawal from the family network. Older adults who perceived to be struggling financially viewed themselves as a burden where they experienced excessive guilt and shame. This contributed to their increased levels of psychological distress.

“My son has to consult his financial advisor (referring to son’s wife). My other son does not give me a single cent. My husband will not allow me to ask my son for money” Female, 77. Other.

#### Financial Strain

This subtheme highlighted the constraints of having no financial support systems from family and thus having to be dependent on either a) working beyond retirement age b) government support or c) support from voluntary services. Being reliant on non-familial support was reported as being particularly distressing in our sample.

“When money is not enough, the church gives me a donation each month”

Female, 65. Indian

#### Financial Independence

In contrast, those older adults who highlighted having *financial independenc*e highlighted the positive impact it had on their psychological health. They stipulated it enhanced their self-esteem and self-worth by feeling they were not dependent on others. They also identified the positive impact this had maintaining family harmony.

“You cannot be dependent on your children. They have their own family… I can manage money- I don’t like to borrow from people or owe people money, if I have less money, I spend less. Female, 70, Chinese.

#### Adaptation and Self-Management of Psychological Health

Three key themes were identified as adaptation and self-management methods when older adults were faced with increased levels of psychological distress. This included: avoidant coping strategies, finding meaning in life by helping others (‘it feels good to do good’) and power of prayer.

### Avoidant Coping Strategies

The first theme highlighted a series of ‘avoidant coping strategies’ which were a common form of adapting and managing emotional worries or at times of increased psychological distress. Examples of these avoidant coping strategies were highlighted within the following subthemes.

#### Supressing Worries

Older adults often tried to maintain regulation of their emotional and cognitive state (the way they think about the way they feel) by ignoring their problems and choosing not to share any worries with others. One participant in particular shared that she was aware the negative impact emotional worries had on her mood but felt sharing her problems with others would not solve anything. The long-term impact of suppressing one’s problems was shown to increase levels of sadness and left older adults with feelings of loneliness. Many older adults reported that suppressing their worries was a method taught to them as young children and the only method they knew to help cope with negative thoughts. The long-term impact of suppressing one’s problems was shown to increase levels of sadness and left older adults with feelings of loneliness. Many older adults reported that suppressing their worries was a method taught to them as young children and the only method they knew to help cope with negative emotion related thoughts.

“Never think of troubles, as troubles can trouble you” Female, 70 Chinese.

#### Retaining Face

This subtheme dominated much of the older adults narrative when asked how they coped with emotionally related stressors. Retaining face in Asian cultures refers to the prevention of embarrassment at all costs. ‘Face’ is important in Asia and is at the core of the persons being which was commonly reflected in our sample particularly amongst the Chinese Singaporeans. The extract below explains the concept of retaining face which highlighted the cultural expectation of not sharing emotional worries with others. By not disclosing their emotional problems they believed it reduced the possibility of shame, embarrassment or ‘gossiping’. This lack of trust in others to share emotional problems and worries was commonly reported not just outside of their social circles but also within their close knit family too. The three extracts below show examples of significance of retaining face in relation to emotional worries.

“What is the use of talking to others about your life? It will only ruin your reputation, you can only know others face but not their hearts” Female, 74, Chinese.

“I don’t talk much with my children, I don’t joke with my children, very seldom talk. I don’t want to give them pressure. Male. 66. Chinese.

“Don’t be sad, don’t be angry, you must self-console, you cannot depend on other people. Female, 70. Chinese.

Our findings highlighted many older adults were very concerned about ‘losing face’ which means losing the respect of others. The difference between this subtheme and the theme of suppressing worries was for many older adults sharing emotional problems was described as a weakness in one’s character.

#### Distraction

The consequences of not sharing problems with others meant that many older adults would use several distraction techniques as coping mechanisms during times of emotional distress. This included engaging in hobbies, watching television but for some participants sleeping excessively was described as a method of distraction and escapism.

“I sleep, and sleep and sleep, why? If I don’t sleep I think so much, so might as well sleep… because of heart pain (referring to emotional pain not physical) people don’t know if I think a lot my heart pains and I cannot breathe, that is why I do not want to think” Female, 74. Indian.

#### It Feels Good to Do Good

A second key theme used as a form of adapting and managing emotional worries at times of increased psychological stress was to help others. This included helping their family members, friends, neighbours as well as people within their communities. This was reported as having a positive impact on their psychological health and many reported that it was able to give them meaning and purpose to their lives as they felt they were at an age where they are unable to work or provide for their families.

Helping and sharing significant concern for others allowed some older adults to feel better about themselves. It also gave many the motivation to look after their own health especially if they were providing care to someone close to them e.g. family member, partner or close friend. They described how caring for others allowed them to put their own lives into perspective and they held an underlying belief that if you do good, good will also come to you.

“Be happy, make others happy, that makes me happy”. Female, 61. Chinese.

#### Power of Prayer

The ‘power of prayer’ was the third dominating theme when older adults were asked specifically about their adaptation and self-management to physical and psychological worries. Participants highlighted that the power of prayer stimulated emotions such as hope and optimism, and was utilised as a common method of coping. For many religion, in particular imparted a spiritual outlook, resilience and acceptance of their ‘problems’ where relegating health needs and emotional worries were transferred to a ‘higher power’ and thus something that had become beyond their control. This belief in a higher power enabled participants to overcome difficult times but also helped them face the ageing experience with an optimistic and resilient attitude.

“Modern medicines are to contain not to cure, (repeats) what cures is this (refers to

Quran), this is the strongest medicine that can cure everything. If it’s God’s will you can fight it out”. Male, 73. Malay.

“Sometimes when there is pain, I pray, then I feel relieved”. Female 66. Chinese.

The power of prayer stimulated faith, helping one find meaning, especially with regard to declines in one’s health.

### Perceptions of Social Health

Social health was conceptualised as an individual’s perception of their social relationships, social activities and their social environment. Two main themes were raised when older adults spoke of their social health. These included; Social connectedness and eating together.

### Social Connectedness

**Fig. 2:**
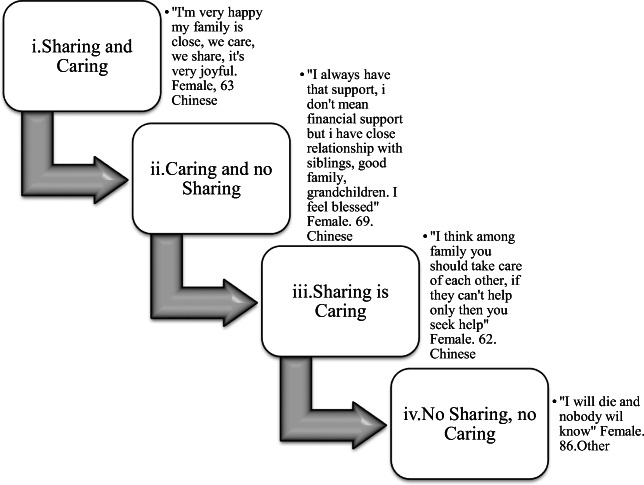
Taxonomy of social relationships amongst Asian older adults in Singapore, (n = 40). **(i). Sharing and Caring:** These older adults’ believed they were not only financially supported by their family, but also emotionally. They highlighted the closeness of the family, with regular contact and activities but also willingness to share health and more significantly, emotional problems with. **(ii). Caring and No Sharing,** this type of relationship existed amongst the older adults who had a supportive family network, but there was very little family support in terms of financial or practical help. This was not because the family didn’t want to help, but more due to financial or practical constraints (e.g. living abroad, low income etc.). **(iii). Sharing is Caring,** represented a group of older adults who were still very satisfied with their family relationships and still felt as supported, but the support they talked about was financial or practical help. When asked about emotional support, Elder’s within this subtheme felt it was inappropriate to share emotional problems with their family or with others. Interestingly, this was the most common form of relationship reported amongst the older adults. **(iv). No Sharing, No caring.** This theme was reflected in a small group of older adults, who had no social support, and thus had very little caring or sharing. These older adults reported ‘no caring and no sharing’ highlighting feelings of loneliness and social isolation

Figure 2 presents a taxonomy of social relationship perceptions in older adults. What the figure demonstrated was the different ideals older adults held regarding social relationships.

In addition, participants also highlighted a shift in social connectedness where many discussed the different expectations they had from their families. Quality of support was now more important to them then quantity. This was reflected in the theme Eating Together.

### Eating Together

Older adults perceived eating together with family and friends as an indication of the quality of their social relationships. Many reported the importance of sitting down and sharing meals with family members and friends and how this was more than ‘eating practice’ but was regarded as an indication of ‘caring’ as it allowed ‘quality’ time to be spent with those close to them. Thus, eating together went beyond the notion of sharing food and became representative of caring and social closeness. For many older adults, the practice of eating together also maintained the ideology of family and closeness.

#### Adaptation and Self-Management of Social Health

For older adults in Singapore the key theme of adaptation and managing their social health was reflected in the main theme of social participation.

### Social Participation

This theme highlighted the importance older adults placed on being socially active, particularly as they aged. Older adults highlighted how as they became older opportunities for socialisation began to decline particularly due to their declining health.

If I have the interest [to take part in social activities] it'll depend on my legs, whether they are obedient’. Male, 65. Chinese.

However for some older adults they highlighted the significance of being active within their local community. This was highlighted in two subthemes: Active community based participation and religious participation.

#### Active Community Participation

The role of active community participation served two key purposes for older adults. Firstly, older adults identified the positive impact of active community participation particularly when it involved helping others. In helping others they believed they still had purpose despite their age. Mr. C (76, Chinese) discussed how he engaged in exercise programmes organised by his local community centre. In addition, he was involved in a local palliative care hospital wellness programme. Secondly, engaging with others was perceived as something that would help keep them ‘healthy’ both mentally and physically. The case of Mr. P, (74, Malaysian) who identified himself as having a ‘very active social life’ serving in several community-based committees- “all these activities keep me healthy”. However, for some older adults, active social participation was difficult due to family commitments which were always prioritised.

“Taking care of grandchildren keeps me busy, no time for social activities, I have less freedom to make friends”. Female, 62. Malaysian.

#### Religious Participation

Religious affiliation and practice was frequently reported in our sample with religious affiliations ranging from Christianity, Buddhism, Taoism, Muslim, and Hinduism. Despite religious affiliation, older adults identified the positive impact engaging in religious activities had on their social health. They highlighted how being part of a religious group allowed them to be around people who shared similar beliefs and they felt a sense of belonging and purpose. Also, in some cases weekly attendance to a place of worship i.e. church, mosque or temple were the only interactions with people outside of their family, thus religious engagement extended to not only individuals through religious practice but also as a form of social engagement. It brought people together in and outside the place of worship. Madam S, (Indian, 75) shared how meaningful she found being at the temple, it not only allowed her to pray but it also allowed her to socialize and meet with her friends regularly. She highlighted how she attended religious studies followed by ‘snacks’ and ‘chit chat’ with friends.

## Discussion

The aim of this study was to examine the perceptions of physical, psychological and social health of older adults within an Asian context (Singapore). Furthermore, we wanted to explore and understand the adaptation strategies older adults pursued to achieve positive health. In this discussion we highlight our key research findings, discuss the limitations of our study and discuss directions for future research.

### Perceptions and Adaptations to Physical, Psychological and Social Health: Key Findings

The resilient attitude towards older adults ageing experiences and physical ageing process was commonly reported within our sample. Despite older adults suffering from multiple health conditions (for example, diabetes, arthritis, COPD) they associated their ability to engage in physical exercise or in social activities as a positive step towards being healthy and what would be considered as ageing successfully. Our findings further support a recent study by Tkatch et al. (2017) who also showed this disconnect between objective and subjective health of older adults. They found older adults who were objectively categorised with multiple chronic conditions described themselves as being healthy and active. The clinical implications of such findings are that service providers should allow older adults to share their views of their physical health and not be solely guided by objective health measures. Combining patients perceptions of their health with objective measurement will allow for a more person centered care approach.

Another key finding from this study related to the perceptions of psychological health amongst Asian participants. Perceptions of psychological health were dominated by the status of their relationships with their family, spouse and extended social networks. Harmony in particular was of great importance, where peaceful and happy interactions among family members but also friends and neighbours was the goal for many participants ‘happiness’. These themes were not dissimilar to a recent study conducted by Ingersoll-Dayton et al. (2001) where Thai older adults also identified harmony, interdependence, acceptance, respect and enjoyment as five key dimensions that defined their psychological wellbeing. Their study showed harmonious relationships had a positive impact on Thai older adult’s psychological wellbeing. Our findings also supported this concept and thus provides further evidence suggesting that measuring family, social and spousal harmony may assist in more accurate measurement of older adults’ psychological wellbeing and health, particularly when measuring this construct in older adults within Asia. A comparative study examining individualist vs. collectivist perceptions in older adults would help further corroborate if these differences are culturally specific and a possible direction for further qualitative investigation.

Financial strains also dominated the influence on older adults psychological health and wellbeing. They reported feeling conflicted with wanting to have financial independence but often having no choice but to be dependent on family to provide financially for them. This created feelings of being a burden and resulted in older adults experiencing excessive guilt and shame which they believed contributed to increased levels of psychological distress. Our results showed that this was particularly evident amongst our Chinese Singaporean sample. One explanation of these increased feelings of burden, shame and guilt in this sample are the perceptions regarding the traditional Confucian concept of filial piety. Chan and Lim (2004) highlighted with the increase in industrialization and urbanization in China, Taiwan, Japan, and Hong Kong there have been fundamental changes to the structure of the family and despite the fact that filial piety is an important socialisation goal in many Chinese societies, social conditions are changing and have come to redefine children’s obligations to their parents (Chan and Lim 2004; Cheng and Chan 2006). These changes in social expectations may also be the case in Singapore as our findings highlighted dependency on children for financial support and security was in some cases their sole option. As the population continues to age worldwide this may become an increasing issue and future health policies should be aware of the negative psychological impact reliance on family may have for those whose families are unable to provide financial assistance. This is an important area for future investigation.

The process of adaptations to psychological health were dominated by themes the ‘*power of prayer’ and ‘it feels good to do good’*. These adaptation methods were not only for times of increased emotional/psychological distress but older adults utilised these coping mechanisms at times of i) poor physical health functioning (physical health) ii) during increased stressful life events (psychological health), iii) to facilitate better social health (social health). Participants described how prayer and faith stimulated emotions such as hope and optimism. Many shared the belief that a ‘higher power’ was responsible for the consequences of one’s health and wellbeing in which they found comfort. Our findings also highlighted how religious institutions such as churches, temples and mosques were more than often a focal point for interaction, exchange and support. Moreover these institutions were the place of worship where many social interactions happened, friends met, families gathered, and supportive activities took place. This raises the question regarding the mechanisms that drive the positive impact of religion/spiritual coping and health.

The theme ‘power of prayer’ characterised religious and spiritual coping mechanisms which is not surprising given our ethnically diverse sample who identified their religious identity as Muslim, Hinduism, Buddhism and Christianity. Similarly, the spiritual underpinnings of the theme ‘it feels good to do good’ was described by older adults as finding meaning in their self-worth by helping others, particularly as they were ageing. Many older adults found engaging in altruistic behaviours such as helping others either through practical forms of help, financial or emotional improved their own wellbeing. Implications of such a finding highlights how future interventions aimed at improving older adults wellbeing may wish to incorporate elements of altruism which does not necessarily require any religious or spiritual affiliation. Post (2015) found a relationship between altruism and ‘happiness’ where engaging in altruistic behaviours resulted in feeling hopeful, happy, and good about oneself as well as feeling more energetic and connected. Thus, the psychological benefit involved in helping others may be more desirable for older adults and when designing interventions for older adults, simply helping others within voluntary programmes could be a factor that contributes to their emotional wellbeing.

In relation to social health, the perception of social connectedness was fundamental to Singaporean older adults. There were conflicting views on being dependent on their younger family members to meet their needs. The taxonomy of social relationships (Fig. 2) represented this complex interconnectedness reflecting the conflicting values regarding financial and practical forms of support. Older adults were appreciative of having practical forms of support but there had become a shift in the expectation of this form of help. The quality of social support was more important where the practice of eating together maintained the ideology of ‘family’ and closeness. The theme eating together went beyond the notion of sharing food and became more representative of caring and social closeness where older adults perceived this behaviour as a form of ‘good social support’.

In summary our findings showed how perceptions of physical, psychological and social health was dominated by the importance placed on harmony and social connectedness with others. These areas stimulate further research and represent the changing times of interdependence needs of Asian older adults. It also highlights potential constructs and dimensions that should be considered when examining psychological and social health in older adults within an Asian context. For example, the construct of harmony highlights the significance of measuring not only family harmony but also perceptions of financial harmony. These two constructs were the two main themes highlighted as causing increased worry and psychological distress. In addition, measurement of social health should go beyond the scope of simply measuring quantity of support. For example, measuring frequency of eating together with family may be a significant indicator into older adults’ perceptions of their social health.

#### Strengths and Limitations

A strength of our study was that we were able to recruit three ethnic groups (Indian, Malay, Chinese) with diverse religious beliefs. This gave an insight into perceptions within these three different ethnic groups; however, we did not see any key differences in emergent themes within these ethnicities. All participants identified themselves as Singaporean, despite their ethnic heritage and thus may explain the similarities in the emergent themes, however, we must highlight the possible bias in the emergent themes as our sample was 65% Chinese Singaporean, thus many themes may have been embedded in Chinese collectivist values. Although we were unable to identify any key differences in the themes raised across ethnicities future studies may wish to examine ethnicity and religious affiliation to account for the possibility of cultural differences in perceptions of physical, psychological and social health.

Another key strength of this study was the ability to conduct interviews in language preference of participant. However, our topic guides were constructed in English using English terminologies and thus we must be aware of potential bias due to back translation where descriptive terminology in one language may not be have the same literal meaning when transcribed in English. However, all transcripts were matched against the audio with disparities in language fully discussed with the bilingual research team.

Regarding methodology, we used a door knocking sampling methodology thus we must acknowledge that the ‘hard to reach’ or most vulnerable older adults who chose not to answer the door may have been missed and may raise the possibility of some participant selection bias. Also, the predominance of a female sample may have impacted results, however we did not observe any key gender differences in the emergent themes.

With regard to the rigour of qualitative findings the sample was carefully selected to address our principle research question with analysis conducted systematically following several stages of interpretation to reach the final themes. To reduce researcher bias constant comparisons and discussion of themes was undertaken with research team members.

## Conclusions

In conclusion, this study highlighted the perceptions of ageing within a diverse Asian context and identified several mechanisms of adaptation and self-management in the face of these social, physical and emotional challenges. Understanding the underpinnings of how older adults perceive the ageing process and how they adapt to such changes can allow us to design better measurement tools for the future ageing population. We propose this should include older adults’ perceptions of health particularly acknowledging psychological and social resources, coping mechanisms and the cultural contexts. Awareness of cultural nuances in older adults’ perceptions of health will help develop more targeted intervention programmes which can assist with the global ageing phenomena.
